# Immunosenescence and interactions for lymphocyte deep immunophenotyping

**DOI:** 10.1186/s12967-024-05883-4

**Published:** 2025-03-07

**Authors:** Kai Guo, Xiaoran Feng, Mingting Peng

**Affiliations:** 1 National Center for Clinical Laboratories, Institute of Geriatric Medicine, Chinese Academy of Medical Sciences, Beijing Hospital/National Center of Gerontology, No. 1 Dahua Road, Dongdan, Beijing, 100730 P.R. China; 2 National Center for Clinical Laboratories, Chinese Academy of Medical Sciences & Peking Union Medical College, Beijing, 100730 P.R. China

**Dear editor**

Immune cell subsets and deep immunophenotyping are critical for diagnosing chronic and infectious diseases. While the clinical significance of single immune cell type has been studied extensively, relatively little is known regarding immune interactions. Immune responses are dysregulated during aging, which manifests as a moderate increase in circulating inflammatory mediators, and alterations in the proportion and functions of immune cells [1]. Aging is accompanied by systemic chronic inflammation, a phenomenon known as “inflammaging”, which is at the core of most chronic diseases in the elderly and may be the underlying cause of reduced cellular responsiveness [1, 4]. The causal relationship between immunosenescence and age-related chronic low-grade inflammation remains unclear.

We previously conducted a longitudinal study of 60 healthy individuals (aged between 18 and 59 years old), and collected the samples biweekly for ten consecutive two-week periods for detecting immune cell subsets [2]. There was considerable heterogeneity in immune cell subsets both within and between individuals. Through systems-level analysis that accounts for both within- and between-biological variation, researchers can more effectively quantify various immune cell types simultaneously and infer overarching regulatory principles.

Furtherly, this study explored the interactions for blood cells, especially lymphocyte subsets based on deep immunophenotyping (percentages), Red blood cell counts (RBC), Hemoglobin (HGB), Platelet counts (PLT), age, and Body mass index (BMI) through systems-level analysis of 43 parameters based on Spearman correlation analyses. As shown in Fig. 1, age correlated negatively with naive T cells and CD56^bright^ cells, and positively with memory T cells, especially central memory T cells (TCM). BMI showed a negative correlation with naive T cells, and a positive correlation with RBC, HGB, B cells, and total memory T cells. RBC and HGB correlated positively with effector memory-expressing CD45RA (TEMRA) CD4 cells and negatively with the CD4/CD8 ratio. Natural killer T (NKT) cells negatively correlated with naive T cells, positively with CD8^+^ and memory T cells, and negatively with the CD4/CD8 ratio. PD-1^+^cells positively correlated with memory T cells, especially the effector memory T cells. Moreover, the CD4/CD8 ratio was positively correlated with TCM cells and negatively with TEMRA cells. Additional details on cellular correlations can be found in Fig. 1.

As previously studied, inflammaging mainly manifests as specific changes in lymphocyte counts and functions, which may reflect the “immune age” of an individual [3]. We observed a decline in naive T cells and CD56^bright^ cells, along with an increase in memory T cells with aging, which is consistent with previous findings [4]. Additionally, while this study found only a very weak positive correlation between PD-1^+^ cells and age, the observed stronger positive association between memory T cells positively correlated with PD-1^+^ cells provides supporting evidence, as PD-1^+^ cells are known to play key roles in immunosenescence. Although the CD4/CD8 ratio generally tends to increase with age, we did not observe any positive correlation in this study. In addition, there was no significant correlation between age and some cell subsets. Those results align with the notion that aging does not equally affect all parts of the immune system. That is, age is not the only factor influencing immunosenescence. For instance, populations with low BMI generally showed significantly reduced CD4^+^ T cell function. Moreover, while high BMI might temporarily boost lymphocyte function, it also correlates with chronic inflammation, potentially reducing immune potential [5]. Our findings showed a positive correlation between BMI and memory T cells, and a negative correlation with naive T cells, supporting previous conclusions. BMI also showed a significant positive correlation with nutrition-related markers such as RBC and HGB, which correlated positively with TEMRA cells and negatively with the CD4/CD8 ratio, suggesting that nutritional status may significantly influence the proportion of immune cells. Therefore, emphasizing the balance between the current function and the potential of immune cells may be crucial for maintaining immune health, given the complexity of the immune system, which is influenced by various factors.

Altogether, our findings further confirmed that immune responses are dysregulated during aging, and also provided new evidence into the interactions for deep immunophenotyping in major cell subsets. Further in-depth research and confirmation are crucial, including expanding the sample size and exploring underlying mechanisms.

**Fig. 1:**
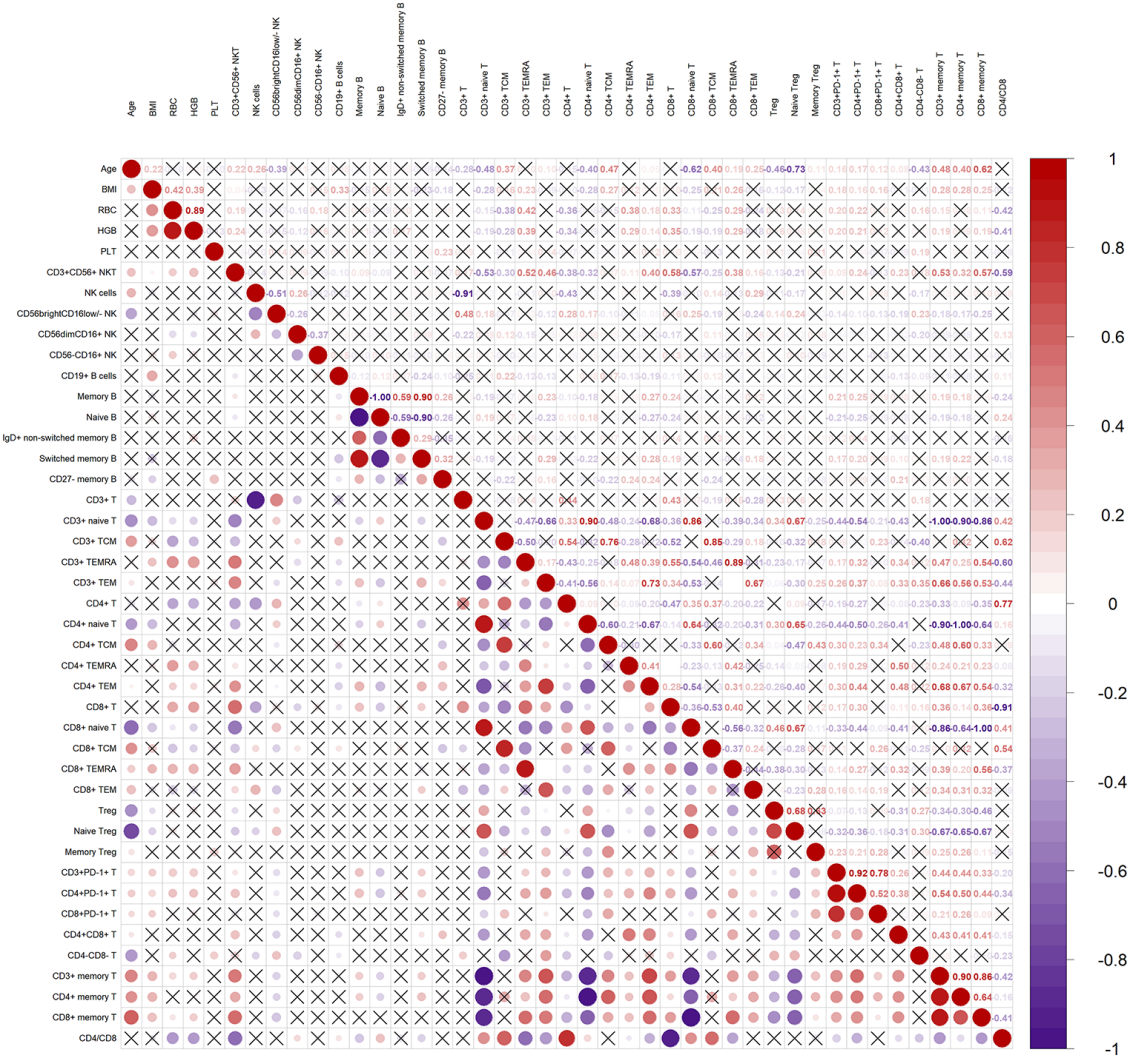
Correlation analysis of lymphocyte subsets based on deep immunophenotyping

## Data Availability

The datasets used and/or analysed during the current study are available from the corresponding author on reasonable request.
